# Heat-Induced Structural Evolution of Coconut Globulin and Its Techno-Functional Properties

**DOI:** 10.3390/molecules31203613

**Published:** 2026-10-10

**Authors:** Xiaoyan Liu, Fuming Deng, Yufeng Zhang

**Affiliations:** 1 Hainan Engineering Center of Coconut Further Processing, Coconut Research Institute, Chinese Academy of Tropical Agricultural Sciences, Wenchang 571339, China; 2 College of Modern Agriculture and Bioengineering, Shangluo University, Shangluo 726000, China

**Keywords:** coconut protein, plant-based food ingredients, protein aggregation, techno-functional properties, structure–function relationship, thermal processing

## Abstract

The heat-induced aggregation behavior of coconut globulin at pH 2.0 and 85 °C was investigated over a continuous heating period of 0–24 h, with a focus on the temporal evolution of protein structure and corresponding changes in techno-functional performance. The aggregation process exhibited three distinct stages: initial unfolding and dissociation (0–6 h), formation of β-sheet-enriched structures (6–12 h), and subsequent over-aggregation (12–24 h). Surface hydrophobicity reached its maximum at 20 h, foaming capacity peaked at 12 h, and emulsifying activity achieved its optimal value at 10 h. These time-dependent functional variations were closely correlated with progressive conformational rearrangement, peptide chain degradation, and interfacial structural changes. The results establish a time-resolved structure–function correlation for coconut globulin under acidic heating conditions, offering practical guidance for the processing of coconut protein as a plant-based functional ingredient.

## 1. Introduction

Plant-based foods are increasingly recognized as key components of sustainable diets, driven by both environmental concerns and consumer demand for healthier, more ethical food choices [1]. Among diverse plant sources, coconut (*Cocos nucifera* L.) has garnered particular attention as a versatile food raw material, with global production exceeding 62 million tons per year [2]. Fresh coconut meat contains approximately 40.9% water, 3.8% crude protein, and 35.2% fat [3], positioning it as a promising source of plant-based ingredients for the food industry. However, the successful integration of coconut into plant-based food products requires a thorough understanding of how its protein components respond to processing conditions and how these responses affect final product quality.

The major protein component in coconut is globulin, accounting for 60–75% of total protein [4]. While this globulin fraction has been reported to exhibit biological activities relevant to human health [5], its direct application in food formulations is constrained by inherent structural limitations. Specifically, the insufficient amphipathic domain distribution of native coconut globulin results in poor oil–water and air–water interfacial activity compared to commercial whey and soy proteins, leading to unsatisfactory emulsifying and foaming performance [6]. Given that protein functionality is fundamentally governed by molecular conformation, controlled processing strategies that modulate protein structure offer a promising route to enhance the processing suitability of coconut protein ingredients for plant-based food applications.

Controlled thermal and pH treatments offer a versatile means to modulate protein structure, promoting the formation of ordered β-sheet-enriched aggregates with enhanced interfacial functionality [7]. This strategy has been successfully applied to various plant proteins, including soy [8], lentil [9], peanut [10], wheat [11], and pea [12], where heat-induced aggregation at acidic pH was shown to improve emulsifying and foaming properties. For instance, it has been demonstrated that red lentil protein aggregates formed under identical conditions exhibited optimal emulsifying performance after 12–18 h of heating [13]. However, the aggregation behavior of coconut globulin under processing-relevant conditions remains largely unexplored. Given its distinct disulfide bonding pattern and hydrophobic profile, the response of coconut globulin to acid–heat processing may differ significantly from that of legume and cereal proteins, warranting dedicated investigation to optimize its functional performance in plant-based food applications.

To date, no continuous time-series study has characterized the structural evolution of coconut globulin during processing, nor has the relationship between processing-induced structural changes and functional performance been established. This knowledge gap limits the rational design of processing conditions for coconut protein ingredients in plant-based food applications. Therefore, the present study provides a systematic, time-resolved characterization (0–24 h) of coconut globulin aggregation during acid–heat treatment (pH 2.0, 85 °C) using a multi-technique approach encompassing spectroscopic, morphological, physicochemical, and functional analyses. The objective is to elucidate the temporal correlation between structural transitions and techno-functional performance (emulsifying and foaming properties), thereby providing mechanistic insights for the processing and high-value utilization of coconut protein as a plant-based food ingredient.

## 2. Results and Discussion

### 2.1. Visual Appearance and ThT Fluorescence Analysis

As shown in Figure S1, the unheated CGA solution was clear and transparent with good light transmittance, containing only a small number of natural aggregates. With prolonged heating time, the solution gradually changed from clear to a translucent milky white, with a slow increase in turbidity, corresponding to the unfolding of proteins and the formation of primary oligomers. The increase in aggregate size reduced the light transmittance. Upon heating to 10–12 h, the turbidity of the dispersion increased significantly, presenting a distinctly translucent white appearance with no phase separation, which aligns with the rapid growth of aggregates as indicated by ThT fluorescence. Further heating to 16–24 h resulted in a stable, homogeneous, opaque milky-white dispersion with turbidity reaching its peak. Crucially, no significant precipitation was observed throughout the entire process. This indicates that under acidic conditions, CGAs exhibit good dispersion stability, which is favorable for their potential use as food ingredients.

**Figure 1:**
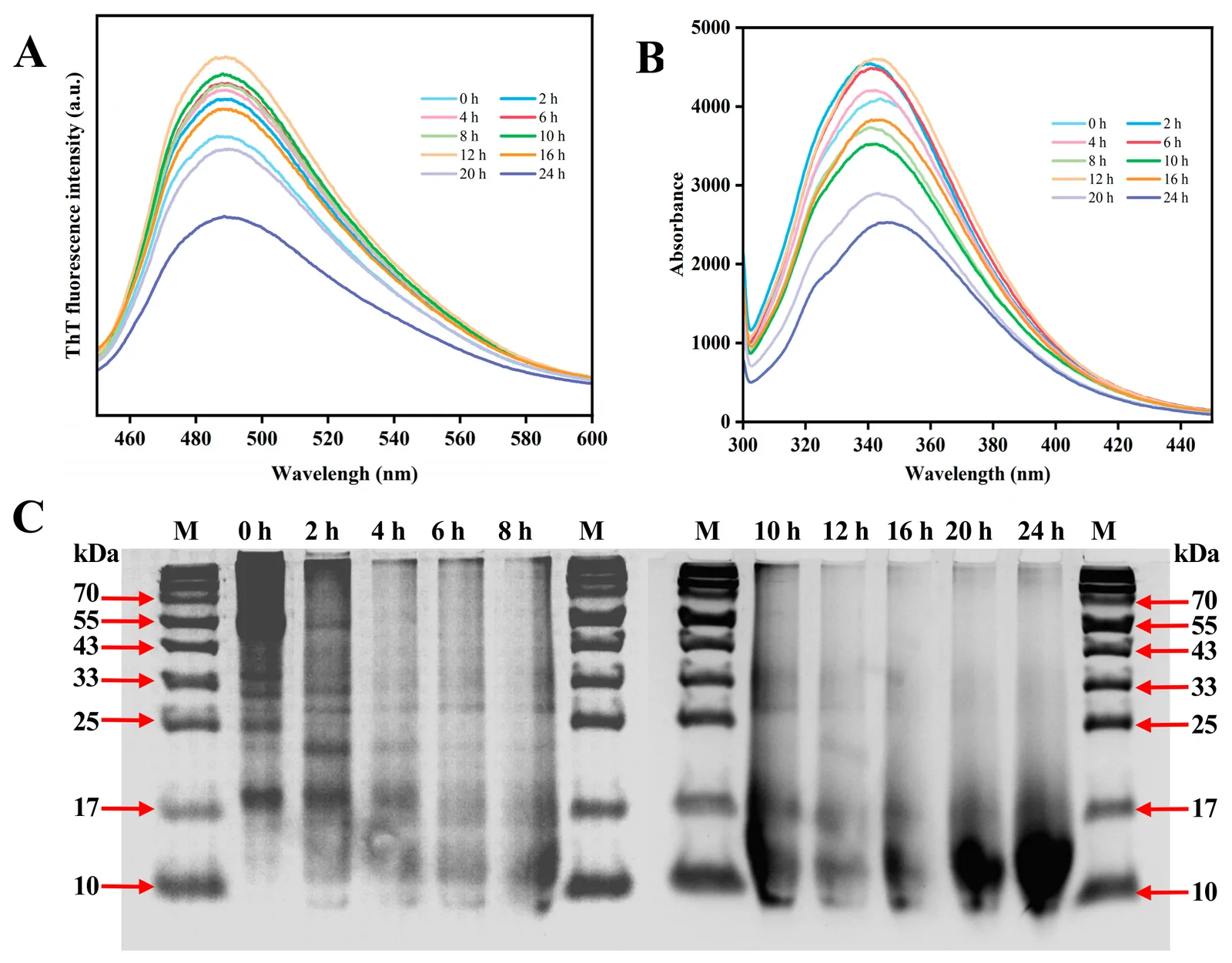
(**A**) ThT fluorescence intensity; (**B**) intrinsic fluorescence; and (**C**) SDS-PAGE profiles of coconut globulin during aggregate formation.

ThT fluorescence enhancement is widely used as an indicator of β-sheet-rich structure formation during protein aggregation [14]. Figure 1A shows the dynamic evolution of ThT fluorescence intensity during the 85 °C heat-induced process of CGAs. Compared to the unheated CGAs (0 h), fluorescence intensity steadily increased during heating from 2 to 12 h, peaking at 12 h. This increase suggests progressive formation of β-sheet-enriched structures during this period. This stage corresponds to a structural rearrangement: acid–heat synergy promotes unfolding of the native conformation, exposing internal hydrophobic regions. Peptide chains then reassemble into β-sheet-rich structures via intermolecular interactions (e.g., π-π stacking, hydrophobic interactions), providing abundant binding sites for ThT [15]. Notably, continued heating from 12 to 24 h resulted in a progressive decline in ThT fluorescence intensity, reaching its lowest point at 24 h. Combined with the FTIR results, which showed only slight changes in the amide I region over the same period, this decline is unlikely to be caused by a loss of β-sheet structure. Instead, it may primarily reflect the burial of ThT binding sites due to lateral association and compaction of aggregates, which reduces their accessibility to the probe.

### 2.2. Fluorescence Spectrum Analysis

The unfolding and refolding states of proteins can be assessed by the intrinsic fluorescence properties of tryptophan and tyrosine. Specifically, the fluorescence spectrum of CGAs formed in a pH 2 system showed a maximum intensity peak at about 350 nm [16]. As displayed in Figure 1B, the fluorescence spectrum of CGAs exhibits significant dynamic evolution during heat-induced self-assembly: emission intensity continuously increases from 0 to 12 h, peaks at 12 h, and then gradually decays from 12 to 24 h. Concurrently, the maximum emission wavelength undergoes a sustained red shift from an initial 340 nm to 345 nm. This biphasic change reveals the microscopic process from protein unfolding to ordered assembly. The initial fluorescence enhancement (0–12 h) primarily stems from thermally induced protein unfolding: internal hydrophobic regions (encapsulating tryptophan/tyrosine residues) are exposed to a polar environment, reducing non-radiative energy transfer and increasing the fluorescence quantum yield. This signifies the formation of monomers and oligomers. Subsequent fluorescence quenching suggests that the formed aggregate structures generate steric hindrance effects on probe fluorescence [17]. Crucially, the persistent red shift phenomenon directly reflects increased microenvironmental polarity at the tryptophan and tyrosine residues, indicating that aromatic side chains are progressively exposed and engage in intermolecular interactions during assembly. This aligns with ThT results: these exposed aromatic side chains, through π-π stacking interactions, constitute the key molecular forces driving the ordered assembly of β-folds [15].

### 2.3. SDS-PAGE Analysis

The molecular weight degradation of CGAs during continuous heating in an environment with pH 2.0 was investigated using SDS-PAGE. Coconut globulin is composed of 86% 11S globulin and 14% 7S globulin [18]. The 11S globulin is a hexameric large subunit (approximately 55 kDa) formed by the polymerization of an acidic subunit (approximately 34 kDa) and a basic subunit (approximately 24 kDa) through disulfide bonds. In contrast, the 7S globulin is a trimer (approximately 55 kDa) consisting of three small subunits with molecular weights of approximately 24 kDa, 22 kDa, and 16 kDa, respectively. As depicted in Figure 1C, the main bands of unheated CGAs were located at approximately 55, 35, 32, 25, and 18 kDa. This may be due to the degradation of some 7S globulin and 11S globulin into small subunits in the environment. Protein bands were observed at the top of the concentrating gel in this lane, which might be attributed to the cross-linking reaction of protein aggregates and the formation of new disulfide bonds, making it difficult for these aggregates to disassemble into monomers even under acidic conditions.

As the heating time was prolonged, the protein bands accumulated at the top of the lane gradually disappeared. Meanwhile, the subunit bands corresponding to proteins with a molecular weight greater than 20 kDa also gradually decreased, while the number of bands ranging from 10 kDa to 17 kDa significantly increased. Previous studies have indicated that heat-treated proteins are susceptible to hydrolysis under acidic conditions, generating low-molecular-weight peptides. These peptides can assemble into oligomers, and with prolonged heating, they can drive the formation of aggregates. However, excessive incubation can disrupt their structure [19,20], which is consistent with these observations. These observations suggest that acid–heat treatment induces partial hydrolysis of coconut globulin into low-molecular-weight peptides. These peptide fragments can further reassemble into larger aggregates upon prolonged heating, which is consistent with the increasing particle size observed at later heating stages. However, excessive incubation may eventually disrupt the aggregate structure.

### 2.4. TEM Analysis

**Figure 2:**
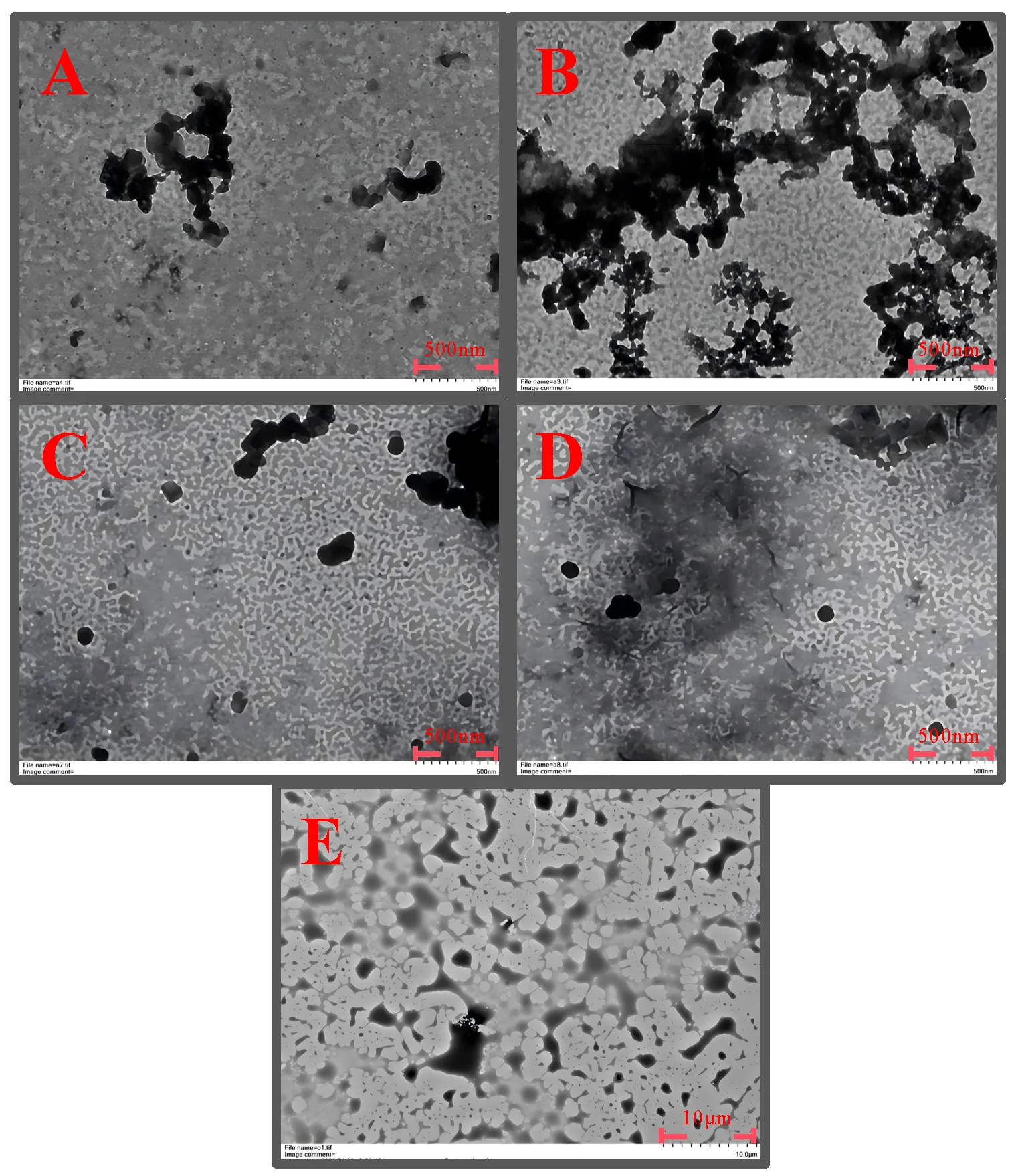
TEM images of coconut globulin aggregates at different heating times. (**A**) 0 h; (**B**) 4 h; (**C**) 10 h; (**D**) 16 h; and (**E**) 24 h.

TEM images of CGAs at different heating times are shown in Figure 2. The unheated CGAs appeared as dispersed irregular particulates. After 4 h of heating, smaller and more fragmented clusters were observed, suggesting partial dissociation of the native aggregates [21]. At 10 h and 16 h, aggregated structures were present, and the aggregates appeared to increase in size and density with prolonged heating. After 24 h, larger and denser aggregate clusters were observed, indicating progressive association of protein aggregates.

### 2.5. Total and Free Sulfhydryl Content Analysis

**Figure 3:**
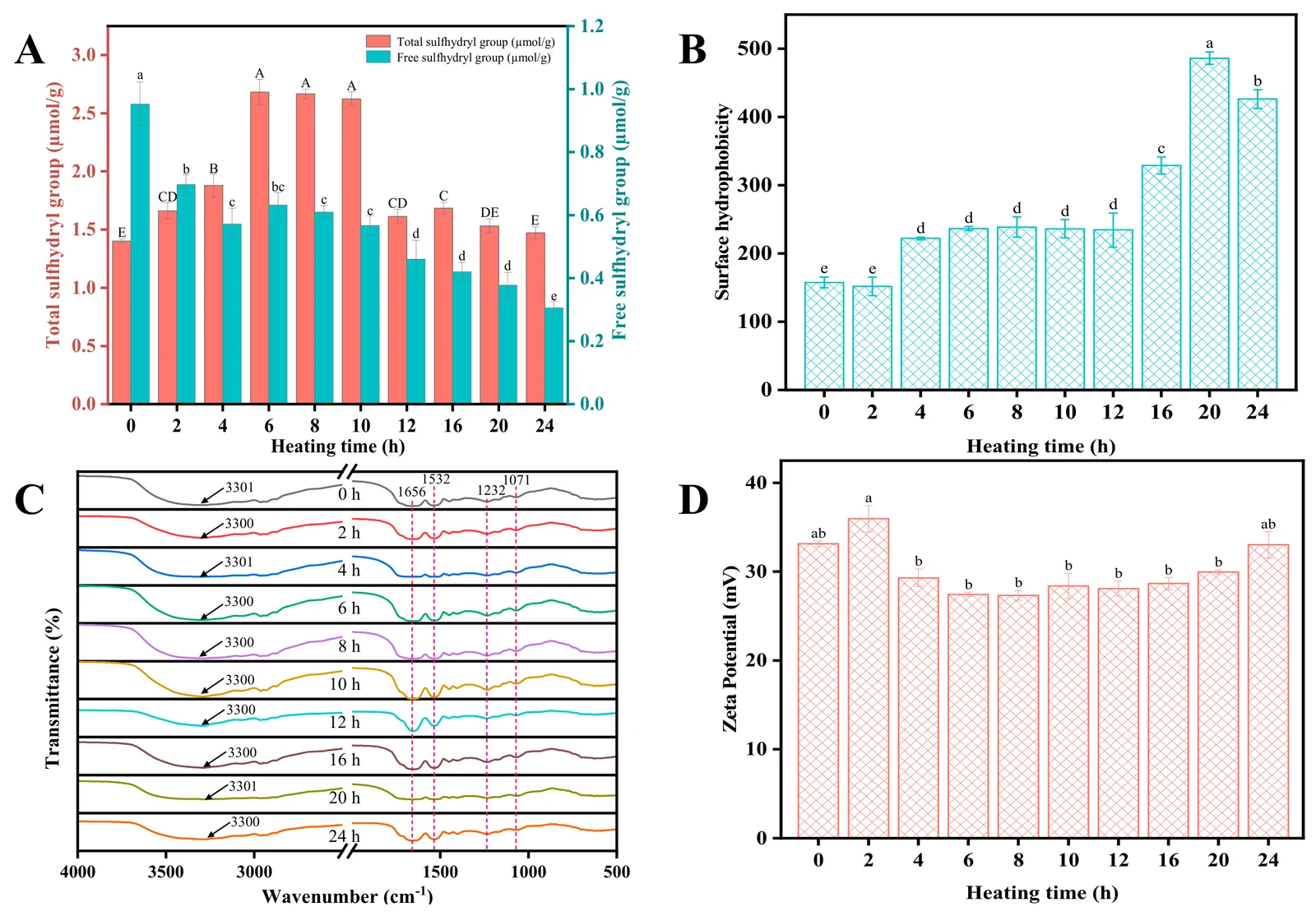
(**A**) Sulfhydryl content; (**B**) surface hydrophobicity; (**C**) FTIR spectra; (**D**) Zeta potential of coconut globulin during aggregate formation. Data are shown as mean ± standard error. Different uppercase letters (A–E) and lowercase letters (a–e) indicate significant differences within each dataset (*p <* 0.05).

Sulfhydryl groups (-SH), as key functional groups maintaining protein structural stability, serve as important indicators for characterizing protein denaturation and aggregation processes based on their content and state changes [22]. As shown in Figure 3A, free sulfhydryl group content remained significantly lower (*p <* 0.05) than that in untreated samples throughout the heating period (2–24 h), likely due to heat-induced consumption of free sulfhydryl groups. This consumption may result from the formation of intermolecular disulfide bonds (-S-S-) or other electrophilic reactions. The change in total sulfhydryl group content exhibits a dynamic process over heating time: during the initial 0–10 h of heat treatment, total sulfhydryl group content exhibited an upward trend. This increase likely resulted from synergistic acid–heat treatment causing severe denaturation of protein tertiary structures. This denaturation exposed “hidden sulfhydryl groups” previously embedded within the molecular interior and inaccessible to reagents, rendering them detectable. However, when incubation exceeded 10 h, total sulfhydryl group content decreased. This may be because, with the formation of extensive aggregate structures, some sulfhydryl groups became physically embedded within dense aggregates [23].

### 2.6. Surface Hydrophobicity Analysis

The surface hydrophobicity (*H*_0_) of CGAs increased significantly with heating time (*p <* 0.05) and exhibited three stages (Figure 3B). During the first 2 h, *H*_0_ showed no significant change, suggesting an initial stage with limited hydrophobic exposure. From 2 to 20 h, *H*_0_ increased rapidly and peaked at 20 h, corresponding to heat-induced unfolding of globulin tertiary structure and exposure of buried hydrophobic residues [6], further amplified by hydrophobic interactions among early-formed aggregates [11]. After 20 h, *H*_0_ slightly declined but remained significantly higher than at early time points. This decrease may result from progressive burial of hydrophobic residues within densely packed aggregate structures, which reduces the accessibility of these sites to ANS probes [24].

### 2.7. FTIR Analysis

FTIR spectroscopy is an effective tool for analyzing protein conformational changes via the amide I band (1700–1600 cm^−1^) [25]. The FTIR spectra of CGAs after different incubation time treatments are depicted in Figure 3C. Heat treatment resulted in vibrations at the wavenumber in the amide A region (3301–3300 cm^−1^), indicating that the protein structure undergoes dynamic changes and the intermolecular hydrogen bonding network undergoes a process of breakage and reorganization [26]. The unheated CGAs exhibited a main absorption peak centered at 1656 cm^−1^, characteristic of a native structure dominated by α-helix and random coil [27,28]. With prolonged heating, this peak showed a slight shift in position, and the relative intensity in the 1630–1620 cm^−1^ region fluctuated across heating times without a consistent monotonic trend, indicating that the local structural environment of this spectral region continued to change during heating. This fluctuation may reflect the dynamic nature of the aggregation process, in which intermolecular interactions are continuously formed and rearranged. However, the FTIR data alone cannot resolve the specific nature of these changes. Therefore, the FTIR results are presented as qualitative evidence for structural reorganization during heating. In future work, complementary techniques will be combined to further elucidate the structural changes underlying these spectral variations.

In addition to the amide I band, the amide II (1600–1500 cm^−1^) and amide III (1400–1200 cm^−1^) regions were also present in the spectra. The amide II band primarily arises from N-H bending and C-N stretching vibrations, while the amide III region is associated with C-N stretching and N-H bending modes. Both regions are sensitive to changes in the hydrogen bonding environment and local secondary structure of the polypeptide backbone. With prolonged heating, these regions exhibited slight fluctuations in peak intensity without a consistent monotonic trend. These fluctuations may reflect continuous rearrangement of intermolecular hydrogen bonds and local conformational adjustments during the aggregation process, consistent with the dynamic structural reorganization indicated by the amide I band and the SDS-PAGE results.

### 2.8. Zeta Potential and Particle Size Analysis

The *ζ* potential increased slightly from 33.13 mV at 0 h to 35.97 mV at 2 h, then decreased to 29.30 mV at 4 h. From 4 to 20 h, *ζ* potential remained relatively stable (27.33–29.97 mV) with no significant differences among these time points (*p* > 0.05). At 24 h, *ζ* potential increased again to 33.03 mV, approaching the value of the unheated CGAs. The initial increase at 2 h may reflect partial unfolding of the globulin tertiary structure, exposing buried charged residues. The subsequent decrease at 4 h is consistent with weakened electrostatic repulsion facilitating aggregation [29]. The relatively stable *ζ* potential from 4 to 20 h suggests that electrostatic interactions were not the dominant driving force during this period, while the increase at 24 h may reflect reorganization of surface charges during aggregate maturation.

Table 1 lists the particle size data for CGAs treated with different incubation times. The unheated CGAs exhibited a *D*_4,3_ of approximately 150 µm and a *D*_1,0_ of 4.28 µm, with a peak diameter of 17.22 µm. The large *D*_4,3_ at 0 h likely reflects a minor population of large aggregates or gel fragments rather than the predominant particle size in the system, since *D*_4,3_ is volume-weighted and even a small number of large particles can dominate the signal. Upon heating for 2 h, *D*_4,3_ decreased sharply to 30.86 µm and *D*_1,0_ dropped to 0.38 µm, indicating dissociation of large aggregates into smaller subunits [30]. The *D*_3,2_ values decreased from 22.06 µm at 0 h to 7.81 µm at 6 h, consistent with an increase in the relative surface area of the particles. A minimum *D*_4,3_ of 15.10 µm was observed at 10 h, with *D*_1,0_ reaching its lowest value of 0.15 µm at 6 h. The peak diameter showed a slight decrease from 17.22 µm at 0 h to 13.46 µm at 4 h, then gradually increased to 17.37 µm at 24 h. At longer heating times (12–24 h), both *D*_4,3_ and *D*_1,0_ increased progressively, reaching 41.80 µm and 8.41 µm at 20 h and 24 h, respectively, indicating reassociation of the subunits into larger aggregates [31].

### 2.9. Foaming Properties Analysis

**Figure 4:**
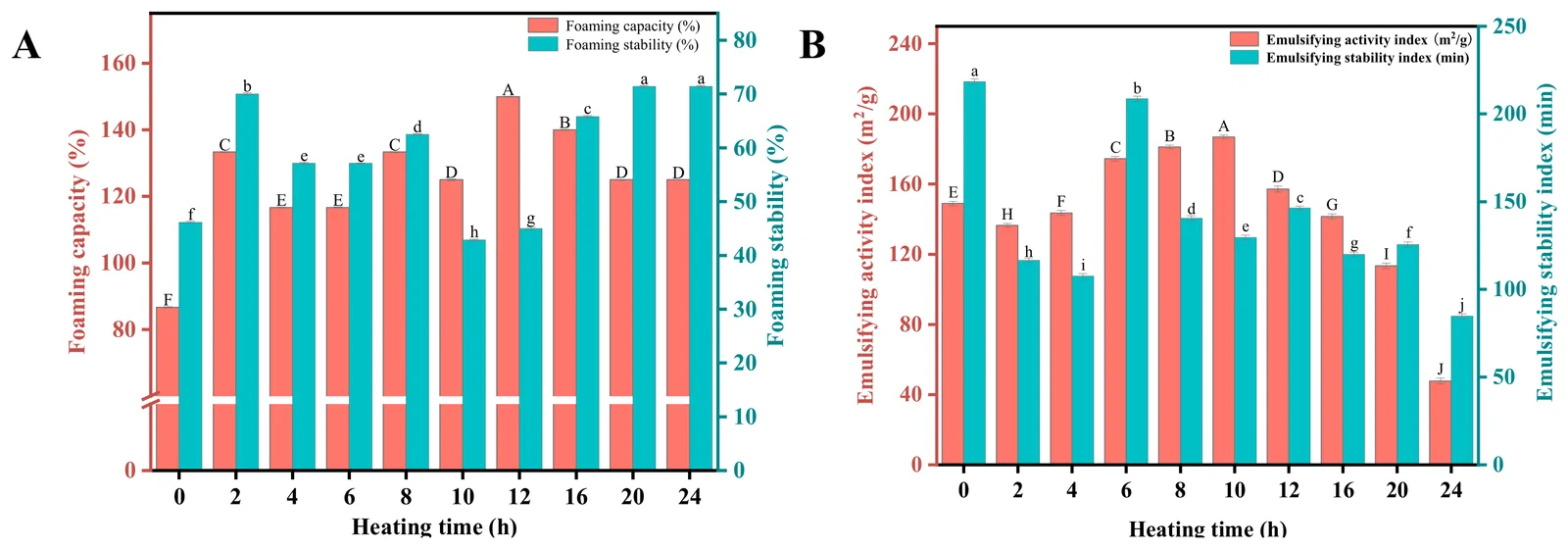
(**A**) Foaming properties and (**B**) emulsifying properties of coconut globulin during aggregate formation. Data are shown as mean ± standard error. Different uppercase letters (A–J) and lowercase letters (a–j) indicate significant differences within each dataset (*p <* 0.05).

Figure 4A illustrates the effect of different heating times on the foaming performance of CGAs. The foaming capacity of unheated CGAs was relatively low (~86.67%). After heat treatment, its foaming capacity increased significantly with heating time, reaching a peak (150%) at 12 h—63.33% higher than the unheated CGAs. This aligns with findings from other protein aggregation studies [32], primarily attributed to the faster adsorption of aggregated proteins onto the air–water interface and the resulting reduction in interfacial tension [29]. Beyond 12 h of heating, foaming capacity slightly decreased (e.g., to ~125% at 24 h), likely due to reduced diffusion efficiency of proteins at the interface caused by excessive aggregation. Foam stability exhibited distinct trends: the unheated CGAs showed a relatively low stability (~46.15%), which significantly improved to ~70% after 2 h of heating. Stability then decreased again, reaching its lowest value at 10 h (~42.86%). Thereafter, foam stability increased from 45.00% at 12 h to 65.79% at 16 h and reached its highest value (~71.43%) at 20 h and 24 h. This variation closely correlates with the dynamic evolution of protein aggregation states [29,32]. Foaming capacity primarily depends on protein interfacial adsorption efficiency. Combined with the particle size analysis data in Table 1, it is inferred that at 12 h, the presence of small and medium-sized particles in the system (*D*_4,3_ = 16.02 µm) provided an optimal surface area-to-volume ratio, enabling rapid interfacial adsorption and bubble encapsulation. Foam stability, however, primarily relies on the mechanical strength of the interfacial film. The well-developed, densely associated aggregate structures formed after 20 h (*D*_4,3_ reaching 41.80 µm) endow the interfacial film with exceptional viscoelasticity and mechanical strength, effectively resisting drainage and coalescence, thus achieving optimal foam stability [20]. The stability decline observed between 4 and 10 h may be attributed to competition for adsorption at the interface among short aggregates, undenatured proteins, and intermediates, resulting in an inhomogeneous interfacial film.

### 2.10. Emulsifying Properties Analysis

Emulsifying properties of proteins, evaluated using the EAI and ESI, are influenced by molecular characteristics and interfacial behavior [33]. As shown in Figure 4B, the CGAs’ EAI decreased at 2 h of heating, possibly due to structural rearrangement without formation of stable emulsifying structures. Thereafter, EAI increased markedly and peaked at 186.90 m^2^/g at 10 h, attributed to progressive hydrophobic exposure reducing interfacial tension and enhancing adsorption [29]. Beyond 10 h, EAI declined, likely due to the formation of large, densely packed aggregates from continued heating [34]. At 24 h, EAI decreased sharply to 47.73 m^2^/g, likely due to the formation of large, densely packed aggregates that have reduced interfacial activity.

For the ESI, unheated CGAs exhibited the highest ESI values. After brief heating of CGAs (2–4 h), the ESI decreased significantly. This trend may be attributed to the formation of small, irregular aggregates by unfolded proteins, which hinder the formation of a continuous, stable interface and thereby reduce the stability of the emulsion. At 6 h, the ESI increased to a secondary peak, likely resulting from protein unfolding and moderate exposure of hydrophobic groups [29]. Thereafter, the ESI decreased again from 8 h onward, reaching its lowest value at 24 h. This decline results from the formation of large, non-uniform aggregates under prolonged heating.

### 2.11. Integrated Structure–Function Relationship Analysis

Based on the changes in ThT fluorescence, FTIR spectra, TEM morphology, and functional properties over time, the aggregation process of coconut globulin at pH 2.0 and 85 °C exhibited three consecutive stages. In the initial stage (0–6 h), the native structure underwent progressive unfolding, as reflected by decreased particle size, increased intrinsic fluorescence, and the appearance of small irregular aggregates in TEM images. In the intermediate stage (6–12 h), β-sheet-enriched structures formed extensively, coinciding with peak ThT fluorescence and optimal foaming capacity. This observation aligns with findings from previous studies on plant protein aggregation, which also noted that moderately sized aggregate fragments exhibit faster diffusion and interfacial adsorption compared to large over-aggregates [13,14]. In the late stage (12–24 h), continued heating led to lateral association and further formation of aggregate structures. Notably, the decline in ThT fluorescence during this period does not indicate degradation of β-sheet secondary structure, but arises from compaction-mediated burial of ThT-binding sites within dense aggregates. This stage was accompanied by reduced emulsifying activity, while foam stability continued to improve up to 20 h. Similar results have been reported that well-developed aggregate structures could form elastic interfacial layers that effectively inhibit bubble coalescence, thus improving foam stability [29]. However, excessive aggregation also leads to the masking of active sites and hindered diffusion, which is consistent with the generally accepted view that oversized aggregates reduce interfacial activity by slowing diffusion and decreasing available surface area [35], resulting in a decline in certain functionalities. These stage transitions suggest that the assembly process involves a balance between β-sheet-enriched structure formation and subsequent over-aggregation and that different functional properties are optimized at different stages of assembly.

## 3. Materials and Methods

### 3.1. Materials

Coconut milk was obtained from a local factory in Wenchang, Hainan. Sodium chloride (NaCl), Thioflavin T (ThT), 1-Anilino-8-naphthalene-sulfonate (ANS), Sodium hydroxide (NaOH), O-phthalaldehyde (OPA), Ammonium persulphate (AP), Coomassie Brilliant Blue G-250, and sodium dodecyl sulfate (SDS) were purchased from Tianjin Hengxing Chemical Reagent Co., Ltd. (Tianjin, China).

### 3.2. Extraction of Coconut Globulin (CG)

The method for preparing coconut milk protein is based on our previous research [36]. The obtained coconut milk protein was mixed with 0.4 M NaCl solution in a 1:10 ratio (*w*/*v*) and stirred for 2 h. The mixture was then centrifuged at 10,000 rpm for 30 min to obtain the supernatant. Next, the supernatant was adjusted to pH 4.0 using 0.1 M HCl and left for 1 h at room temperature. The precipitate was dialyzed for 48 h (dialysis membrane MWCO: 3500 Da) and then lyophilized to obtain the coconut globulin (CG). Finally, the CG was sealed in a nylon bag and placed in a desiccator for subsequent experiments.

### 3.3. Preparation of CG Aggregates (CGAs)

CG aggregates (CGAs) were prepared according to a previously reported method with minor modifications [17]. A suspension was prepared by dispersing the CG in Milli-Q water at 10% (*w*/*v*). The suspension was titrated to pH 2.0 using 2 M HCl after continuous stirring for 2 h, and then placed at 4 °C for 22 h to fully hydrate. Next, the solution was centrifuged at 10,000 rpm for 20 min and filtered through a 0.45 μm syringe filter to effectively remove insoluble proteins. The protein content of the treated solution was determined to be 0.2 mg/mL using the Bradford method. The protein solution was divided into 10 equal portions in 100 mL glass vials and incubated at 85 °C for 0, 2, 4, 6, 8, 10, 12, 16, 20, and 24 h, respectively. The reaction products were immediately cooled with ice water and could be stored at 4 °C or lyophilized for storage according to the subsequent test requirements. Unless otherwise stated, the initial concentration of coconut globulin used for all aggregation reactions was 0.2 mg/mL at pH 2.0, and all subsequent experiments were performed using the heated reaction mixtures directly.

### 3.4. Thioflavin T (ThT) Fluorescence Spectroscopy

The process of CGA was investigated by determining the ThT fluorescence intensity according to a previous method with some adjustments [37]. ThT stock (7.9 mg in 8 mL PBS, pH 7.0) was filtered, stored at 4 °C in the dark, and diluted 50-fold before use. Fluorescence was measured (F-7000, Hitachi High-Tech Corp., Tokyo, Japan) at *λ*_ex_ 440 nm/*λ*_em_ 482 nm (scan 440–600 nm) with 5 nm slit widths at ambient temperature, after mixing 1 mL working solution with 10 μL sample.

### 3.5. Intrinsic Fluorescence

The intrinsic fluorescence of CGA was measured according to a previous method with modifications [13]. The protein sample solutions were transferred into quartz cuvettes individually and then sequentially measured using a fluorescence spectrophotometer (F-7000, Hitachi High-Tech Corp., Tokyo, Japan). The parameters were set to an excitation wavelength of 280 nm, an emission wavelength scanning range of 290 to 420 nm, and both excitation and emission slit widths were kept at 5 nm.

### 3.6. Sodium Dodecyl Sulfate–Polyacrylamide Gel Electrophoresis (SDS-PAGE)

According to a previous method, 15% separating and 5% stacking gels for SDS-PAGE were prepared [38]. The sample solution (0.2 mg/mL) was then mixed with reducing buffer containing β-mercaptoethanol and boiled. After cooling, 8 µL of the mixture, as well as the marker, was taken and spotted in the corresponding wells of the SDS-PAGE gel. The electrophoresis process was carried out at a constant voltage of 80 V until the samples completely migrated to the bottom of the gel. At the end of electrophoresis, the gels were stained using Coomassie Brilliant Blue G-250. They were then decolorized using a decolorizing solution containing 10% acetic acid and 10% methanol.

### 3.7. Transmission Electron Microscopy (TEM)

Microstructures of the CGAs were observed by a TEM instrument (HT-7700, Hitachi High-Tech Corp., Tokyo, Japan). CGAs were uniformly deposited on a 300-mesh copper grid (Ted Pella, Inc., Redding, CA, USA) under ambient conditions. After 5 min of contact, the excess solution was blotted out with filter paper. After the sample on the copper mesh was readily and completely air-dried, microstructural observations of the sample were made using a TEM instrument with an accelerating voltage of 120 kV.

### 3.8. Total and Free Sulfhydryl Group (-SH) Content

The -SH content was determined according to a previous method with minor modifications [39]. To determine total sulfhydryl content, 1 mL sample solution was taken and mixed with 2 mL Buffer I (0.086 mol/L Tris, 0.09 mol/L Glycine, 0.04 mol/L EDTA, pH = 8.0) and 20 µL of Ellman’s reagent. The mixture was allowed to stand for 5 min at room temperature, and then the absorbance was measured at 412 nm. The content of free sulfhydryl was measured using the same method, but buffer II (0.086 mol/L Tris, 0.09 mol/L glycine, 0.04 mol/L EDTA, 8 mol/L urea, pH = 8.0) was used instead of buffer I. The sulfhydryl content was calculated according to the following formula:

$$-SH(\mu mol/g)=\frac{73.53\times D\times A_{412}}{C}$$

where *A*_412_ represents the absorbance measured at 412 nm; *D* represents the dilution factor; and *C* represents the protein concentration in the sample solution.

### 3.9. Surface Hydrophobicity (H_0_)

The surface hydrophobicity was determined by reference to previous methods [40]. Protein samples were diluted with Milli-Q water to 0.05–0.20 mg/mL. Then, 2 mL of each dilution was mixed with 20 μL of 8 mM ANS and incubated at room temperature for 15 min in the dark. Fluorescence was measured (F-7000, Hitachi High-Tech Corp., Tokyo, Japan) at *λ*_ex_ 390 nm and *λ*_em_ 470 nm. *H*_0_ was calculated from the initial slope of fluorescence intensity versus protein concentration.

### 3.10. Fourier Transform Infrared Spectroscopy (FTIR)

The FTIR spectra of the samples were recorded using a Thermo Scientific Nicolet iS10 FTIR spectrometer (Thermo Fisher Scientific, Madison, WI, USA). The dried sample powder was mixed well with KBr in the ratio of 1:100 (*w*/*w*) and pressed into tablets. The FTIR spectrometer wavelength range was set to 4000–600 cm^−1^ and the number of scans was 32 times.

### 3.11. Zeta (ζ) Potential and Particle Size

The *ζ* potential of CGAs was measured using a Zetasizer Nano ZS90 (Malvern Instrument Ltd., Worcestershire, UK). For *ζ* potential measurement, the solution was injected slowly into the potentiometric cell, ensuring that the electrodes were well-moistened and no air bubbles were introduced. Three measurements were taken for each sample. The particle size distribution was measured using a laser diffraction particle size analyzer (BT-9300S, Dandong Bettersize Instruments Co., Ltd., Dandong, China) with a nominal measurement range of 0.1–1200 μm [41].

### 3.12. Foaming Capacity (FC) and Foam Stability (FS)

The foaming properties of CGAs were determined according to a previous method with minor modifications [33]. The CGA solutions were homogenized (10,000 r/min, 2 min) with the FJ200-SH homogenizer (Shanghai Huxi Analysis Instrument Factory Co., Ltd., Shanghai, China). The solution, after sufficient stirring, was quickly transferred to the measuring cylinder. The total amount of foam was recorded at 0 and 30 min intervals after homogenization. FC is defined as the ratio of foam volume measured immediately after homogenization to the initial volume of CGAs, while FS represents the ratio of foam volume after 30 min of standing to foam volume after initial homogenization.

### 3.13. Emulsifying Activity Index (EAI) and Emulsifying Stability Index (ESI)

The emulsifying properties of CGA solution were measured with a few modifications according to a previous method [42]. Briefly, 4 mL of CGA solution was mixed with 8 mL of coconut oil at 10,000 r/min for 3 min with the FJ200-SH homogenizer (Shanghai Huxi Analysis Instrument Factory Co., Ltd., Shanghai, China). Subsequently, at the end of homogenization, at 0 and 60 min, 100 µL of the emulsion was aspirated from the bottom of the tube and mixed thoroughly with 5 mL of 0.1% (*w*/*v*) SDS solution, which was subsequently transferred to a cuvette and the absorbance value of the mixture was measured at 500 nm. The formulas for the EAI and ESI are shown below:

$$EAI(m^{2}/g)=\frac{2\times 2.303\times D\times A_{0}}{C\times \varphi \times \theta \times 1000}$$

$$ESI(min)=\frac{A_{0}}{A_{0}-A_{60}}\times ∆t$$

where *A*_0_ is the absorbance value of the emulsion at the initial moment (t = 0 min) after homogenization; *D* is the dilution factor; *C* is the protein concentration; *φ* is the optical range (0.01 m); *θ* is the volume fraction of the oil phase (2/3); *A*_60_ is the absorbance after 60 min of homogenization; and Δ*t* = 60 min.

### 3.14. Statistical Analysis

Data analysis in this study was performed using IBM SPSS 26 software (IBM Corp., Armonk, NY, USA). Each experiment consisted of at least three replicate determinations of independent samples. One-way ANOVA and Duncan’s test were used to determine significant differences between groups, and different letters were used to indicate significant differences (*p* < 0.05). All images were produced through Origin 2022 (OriginLab, Northampton, MA, USA).

## 4. Conclusions

This study demonstrates that acid–heat treatment (pH 2.0, 85 °C) effectively modulates the structural and functional properties of coconut globulin in a time-dependent manner. The structural evolution, monitored over 24 h, revealed three distinct stages: initial unfolding and dissociation (0–6 h), formation of β-sheet-enriched structures (6–12 h), and subsequent over-aggregation (12–24 h). These structural transitions were temporally correlated with specific functional outcomes: emulsifying activity peaked at 10 h, foaming capacity at 12 h, and foam stability along with surface hydrophobicity at 20 h. These findings establish a processing–structure–function relationship for coconut globulin and provide a mechanistic basis for tailoring its techno-functional performance through controlled heat treatment. Further studies under different pH conditions and in food matrices are warranted to validate practical applicability.

## Figures and Tables

**Table 1:** Particle size distribution of coconut globulin during aggregate formation.

Heating Time	*D*_4,3_	*D*_3,2_	*D*_1,0_	Peak
0	150.07 ± 10.02 ^a^	22.06 ± 0.47 ^a^	4.28 ± 1.55 ^c^	17.22 ± 0.04 ^ab^
2	30.86 ± 1.33 ^bc^	10.67 ± 0.07 ^g^	0.38 ± 0.01 ^e^	15.38 ± 0.21 ^e^
4	31.98 ± 0.46 ^bc^	8.65 ± 0.03 ^h^	0.26 ± 0.01 ^e^	13.46 ± 0.19 ^g^
6	39.76 ± 0.64 ^bc^	7.81 ± 0.05 ^i^	0.15 ± 0.01 ^e^	15.14 ± 0.06 ^f^
8	36.94 ± 1.21 ^bc^	12.39 ± 0.24 ^e^	2.56 ± 0.41 ^d^	15.48 ± 0.01 ^e^
10	15.1 ± 0.47 ^d^	11.8 ± 0.07 ^f^	2.87 ± 0.33 ^d^	15.62 ± 0.04 ^e^
12	16.02 ± 0.28 ^d^	13.71 ± 0.04 ^d^	5.19 ± 0.02 ^bc^	16.16 ± 0.23 ^d^
16	41.04 ± 2.81 ^b^	15.53 ± 0.08 ^c^	6.4 ± 0.05 ^b^	16.84 ± 0.03 ^c^
20	41.8 ± 7.78 ^b^	15.85 ± 0.12 ^c^	6.39 ± 0.04 ^b^	17.11 ± 0.03 ^b^
24	29.53 ± 3.22 ^c^	16.85 ± 0.03 ^b^	8.41 ± 0.34 ^a^	17.37 ± 0.01 ^a^

Values are the mean of three replicates ± standard deviation. ^a–i^ Different lowercase letters indicate significant differences (*p* < 0.05) among samples.

## Data Availability

All data presented in this study are available on request from the corresponding author. The data are not uploaded in publicly accessible databases.

## Supplementary Materials

The following supporting information can be downloaded at https://www.mdpi.com/article/10.3390/molecules31203613/s1. Figure S1: Appearance of CGAs incubated at pH 2.0 and 85 °C for 0, 2, 4, 6, 8, 10, 12, 16, 20, and 24 h.
