# Supplementary material for: Heat-Induced Structural Evolution of Coconut Globulin and Its Techno-Functional Properties
Source: Molecules. 2026 Oct 10;31(20):3613. doi: 10.3390/molecules31203613 (PMC13707485; doi:10.3390/molecules31203613)
Supplement: Supplementary file 1 [file molecules-31-03613-s001.zip › molecules-4584567-supplementary.pdf]

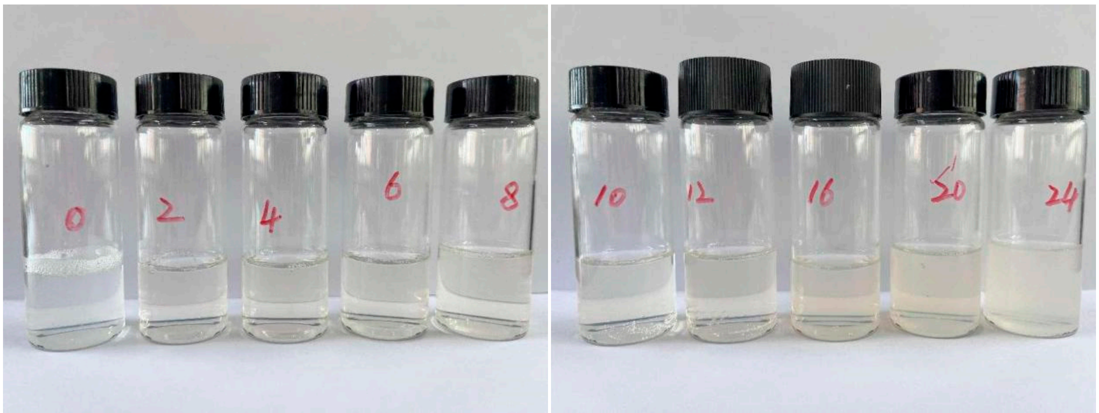

Figure S1. Appearance of CGA incubated at pH 2.0 and 85 °C for 0, 2, 4, 6, 8, 10, 12, 16, 20, and 24 h.
